# Cell-Type-Specific ROS–AKT/mTOR–Autophagy Interplay—Should It Be Addressed in Periimplantitis?

**DOI:** 10.3390/diagnostics14242784

**Published:** 2024-12-11

**Authors:** Mihai Butucescu, Marina Imre, Florentina Rus-Hrincu, Bianca Voicu-Balasea, Alexandra Popa, Mihai Moisa, Alexandra Ripszky, Cristina Neculau, Silviu Mirel Pituru, Simona Pârvu

**Affiliations:** 1 Department of Organization, Professional Legislation and Management of the Dental Office, Faculty of Dental Medicine, “Carol Davila” University of Medicine and Pharmacy, 17-23 Plevnei Street, 020021 Bucharest, Romania; mihai.butucescu@umfcd.ro; 2 Department of Prosthodontics, Faculty of Dental Medicine, “Carol Davila” University of Medicine and Pharmacy, 17-23 Calea Plevnei, 010221 Bucharest, Romania; marina.imre@umfcd.ro; 3 Department of Biochemistry, Faculty of Dental Medicine, “Carol Davila” University of Medicine and Pharmacy, 17-23 Plevnei Street, 020021 Bucharest, Romania; florentina.rus-hrincu@umfcd.ro (F.R.-H.); alesandra17popa@gmail.com (A.P.); mihai-radu.moisa@drd.umfcd.ro (M.M.); alexandra.ripszky@umfcd.ro (A.R.); 4 The Interdisciplinary Center for Dental Research and Development, Faculty of Dental Medicine, “Carol Davila” University of Medicine and Pharmacy, 17-23 Plevnei Street, 020021 Bucharest, Romania; biancavoicu2106@gmail.com; 5 National Institute of Public Health, General Medicine Faculty, “Carol Davila” University of Medicine and Pharmacy, 050474 Bucharest, Romania; simona.parvu@umfcd.ro

**Keywords:** autophagy, periimplantitis, autophagy modulators, ROS, dendritic cells, gingival fibroblasts, macrophages, osteocytes, osteoblasts

## Abstract

Periimplantitis represents an inflammatory disease of the soft and hard tissues surrounding the osseointegrated dental implant, triggering progressive damage to the alveolar bone. Cumulative data have revealed that periimplantitis plays a crucial part in implant failure. Due to the strategic roles of autophagy and its upstream coordinator, the AKT/mTOR pathway, in inflammatory responses, the crosstalk between them in the context of periimplantitis should become a key research target, as it opens up an area of interesting data with clinical significance. Therefore, in this article, we aimed to briefly review the existing data concerning the complex roles played by ROS in the interplay between the AKT/mTOR signaling pathway and autophagy in periimplantitis, in each of the main cell types involved in periimplantitis pathogenesis and evolution. Knowing how to modulate specifically the autophagic machinery in each of the cellular types involved in the healing and osseointegration steps post implant surgery can help the clinician to make the most appropriate post-surgery decisions. These decisions might be crucial in order to prevent the occurrence of periimplantitis and ensure the proper conditions for effective osseointegration, depending on patients’ clinical particularities.

## 1. Introduction

A dental implant insertion is considered healthy when there are no visible clinical signs of inflammation. Normally, the periimplant soft tissues have a coral-pink colouration and firm consistency. Also, there is no bleeding and/or suppuration upon probing, and no marginal bone loss after the initial physiological bone remodelling [1,2,3]. The existing literature has identified and highlighted the main factors that could increase the susceptibility to periimplantitis of an implant insertion site [1,2,3]. These factors are summarized in Table 1. All these factors can seriously affect an individual’s ability to control plaque formation and removal [1,2].

Cumulative data have revealed that periimplantitis plays a crucial part in implant failure [1,2,3]. It has been shown that approximately 30% of patients who have undergone dental implant surgery develop periimplantitis [1,2]. Moreover, it has been claimed that the prevalence of periimplantitis can increase as a function of time [1,2]. Therefore, considering the number of unresolved or recurring periimplantitis cases after five years [1,2], the best clinical solution would be to avoid this condition at all costs. Furthermore, as dental implant use is constantly increasing, new and efficient prevention strategies and therapeutic protocols for periimplantitis are therefore required.

Periimplantitis refers to an inflammatory disease of the soft and hard tissues surrounding the osseointegrated dental implant, triggering progressive damage to the alveolar bone. Experimentally induced periimplantitis studies have revealed that bacterial plaque represents the etiological agent in periimplant diseases [1,2]. However, periimplantitis is a multi-factorial disease, with a very complex inflammatory background occurring in both the soft and hard oral tissues which surround the dental implant [1,2]. Therefore, exploring the important cellular signaling pathways involved in the molecular mechanisms of inflammation will open new ways toward a better understanding of this disease [1,2,3,4].

The complications that usually affect the periimplant tissues are classified as periimplant mucositis and periimplantitis [1,2,5]. Periimplant mucositis is regarded as the preceding step of an infectious process which might evolve toward periimplantitis [1,2,4,5]. In periimplant mucositis, a biofilm-induced inflammatory process is induced, and may progress with different intensities [4,5,6]. The continuous accumulation of inflammatory infiltrate around the implant promotes the advancement of the inflammatory process to the surrounding bone [1,4,6,7,8]. The inflammatory events’ expansion into the alveolar bone might open the way toward periimplant bone loss [1,4,6,7,8].

The crosstalk of autophagy and inflammatory reactions plays a crucial role in the context of both physiological and pathological states [9,10]. Autophagy represents a core molecular pathway, designed during evolution for the preservation of cellular homeostasis [10]. Also, autophagy is essential in inflammatory response regulation. The interplay between autophagy and cytokines should be regarded as a key mechanism involved in the coordination of innate and adaptive immune systems’ activities [9,10,11,12,13,14]. Moreover, nanomaterial-induced autophagy modulation might be regarded as a molecular instrument for achieving anti-inflammatory and pro-osseointegration effects, especially in the case of at-risk patients (such as heavy smokers and diabetic patients). Also, based on the complex role played by autophagy, as either a pro-survival or a pro-death agent [14], further targeting might downregulate the inflammatory responses of fibroblasts and immune cells like macrophages (Table 2). However, for more accurate molecular responses and more efficient clinical results, it should not be forgotten that autophagy is rigorously orchestrated by the AKT/mTOR pathway, and that the communication language between these two vital signaling pathways is written by reactive oxygen species (ROS) [15,16,17,18,19,20,21,22]. ROS represent necessary cellular metabolism products, as they play key roles in cellular signaling, especially in pro-survival signaling [15,16,17,18,19,20,21,22,23].

In order to prepare accurate autophagy-modulating therapeutic molecular instruments, future studies should not focus only on the mechanism of autophagy itself, but should explore the complex molecular context of its regulation by the AKT/mTOR pathway, the molecular translator of extracellular signals. Due to the strategic roles of autophagy and its upstream coordinator, the AKT/mTOR pathway, in inflammatory responses, the crosstalk between them in the context of periimplantitis should become a key research target, as it opens up an area of interesting data with clinical significance.

Therefore, in this article, we aimed to briefly review the existing data concerning the complex roles played by ROS in the interplay between the AKT/mTOR signaling pathway and autophagy in periimplantitis, in each of the main cell types involved in periimplantitis pathogenesis and evolution (Table 2).

According to our knowledge, there are very few studies focused on integrating the information regarding the interplay between the AKT/mTOR signaling pathway and autophagy in periimplantitis, in all the main cell types involved. This integrated information might open the way toward the elaboration of autophagy-modulating instruments for periimplantitis prevention and treatment, targeting specific cell types in accordance with the patient’s clinical landscape and possible risks.

For this narrative review, the PubMed library was searched using the following keywords: periimplantitis, inflammation, Akt/mTOR, autophagy, Akt/mTOR–autophagy interrelations, ROS, oxidative stress, macrophages, dendritic cells, oral fibroblasts, osteoblasts, osteoclasts, osseointegration, dental implant failure, Porphyromonas gingivalis.

## 2. Periimplantitis—Short Molecular Insights

Immunity plays a key role during wound healing and osteogenesis around dental implants [23]. In the first stage after dental implant surgery, inflammation represents a protective mechanism for homeostasis restoration, targeting the injury site and the tissue repair processes [6,9,23].

Periimplantitis is unanimously recognized as a destructive inflammatory lesion which affects the implant’s integration into the surrounding soft and hard tissues, resulting in periimplant pocketing and bone loss [23].

Healthy periimplant tissues normally display many coccoid cells with low levels of anaerobic and Gram-negative species [3]. However, periimplantitis lesions are characterized by increased levels of Gram-negative bacteria, the main species identified being *Porphyromonas gingivalis* (Pg) and *Aggregatibacter actinomycetemcomitans* [3]. Pg is widely recognized as a keystone pathogen due to its ability to manipulate the complex oral microbiome, mediating bacteria cell coaggregation, biofilm formation and oral microbial dysbiosis [3,24].

Some of the most important actors on the inflammation scene are the AKT/mTOR pathway and autophagy, and especially their interrelations. Moreover, with it being known that ROS are essential players in functionalizing the AKT/mTOR–autophagy crosslinks, redox homeostasis and oxidative stress (OS) studies should be regarded as important research targets [15,16,17,18,19,20,21,22].

Cellular ROS are produced by at least four major sources: ROS-generating NADPH oxidase (NOXs), mitochondria, peroxisomes and the endoplasmic reticulum [17,18,19,20,21]. Highly controlled, intermediate levels of ROS, gained during aerobic life evolution, play important roles as short-lived second messengers in cell signaling [18]. ROS have multifaceted properties because they are needed to sustain cellular functions in both health conditions and disease [17,18,19,20,21]. Currently, OS is defined as an imbalance between oxidants and antioxidants, created due to a predominance of oxidants, leading to alterations in redox signaling and control, and molecular damage [20,21]. ROS overabundance triggers an increased oxidant load, which, together with either reduced or unaltered antioxidant protection, leads to oxidative stress [17,18,19,20,21]. Within the affected cells, oxidative stress represents the first step toward pathological changes and, consequently, the destruction of host tissues [19].

It has been claimed that Pg is able to strongly stimulate the host immune system by releasing pathogenic factors like lipopolysaccharide (LPS) molecules and invading periodontal tissue cells [24,25,26]. Inflammatory cells recruited as a result of the host immune response (such as neutrophils in the initial infectious lesion step, macrophages in the early infectious lesion step, and B lymphocytes in the later and advanced infectious lesion step) synthesize and secrete pro-inflammatory cytokines (IL-1β; IL-6 and TNFα), and also, as a consequence of their immune functions, release excessive levels of ROS [27,28]. Moreover, Pg endotoxins induce the release of IL-1β and prostaglandin E2 by monocytes, fibroblasts and macrophages, which are the main actors in the bone resorption stage [29,30].

Macrophage phagocytosis is induced by several cell-receptor–ligand interactions in order to clear pathogens and dead cells from a host. During phagocytosis, ROS (especially O_2_^−^, H_2_O_2_ and radical OH), representing the end products of mitochondrial respiration, cause mainly protein damage, lipid peroxidation and, finally, DNA lesions [31,32,33]. All of these lead to an oxidative imbalance, which induces pro-inflammatory reactions [31,32,33]. These pro-inflammatory responses may evolve into osteoclastogenesis, leading to the bone loss observed in periimplantitis patients [31,32,33]. Although primarily acting as antimicrobial weapons, ROS should be regarded as a “double-edged sword”. These chemical species promote health, as they are involved in the destruction of invading pathogenic microorganisms; however, in the case of overproduction, they may become cytotoxic to host cells [31,32,33]. The OS caused by ROS overproduction, and the imbalance of the cellular redox status, can activate a complex panel of transcription factors, which regulate, in various ways, the expression of specific genes involved in inflammatory cascades. Many studies have outlined the key role that ROS play in the inflammatory process [31,32,33,34,35,36].

Moreover, the overproduction of ROS as a consequence of an overstimulated respiratory burst has been associated with an enhanced expression of pro-inflammatory cytokines [37,38,39,40,41]. These cytokines are responsible for both the indirectly and directly connective tissue destruction, and, finally, the bone resorption events, that characterize periimplantitis [42,43,44,45]. The wide range of matrix metalloproteinases released at excessive levels into the infection site during inflammatory responses, and the reduced levels of collagen synthesis by ROS-affected gingival fibroblasts, make a great contribution to both the promotion of connective tissue and the degradation of the bone matrix [46,47,48,49,50].

However, regarding the positive aspects of the “double-edged” sword image of ROS, these chemical species are considered key autophagy promotors [51]. Also, ROS are regarded as playing a decisive role in the activation of the PI3K/AKT/mTOR pathway, which, in turn, is involved in redox balance regulation [52]. The existing literature outlines an antagonistic relationship between the PI3K/AKT/MTOR pathway and autophagy in the context of inflammation [51,52,53,54,55,56,57,58,59,60]. ROS represent important messengers for ensuring this relationship [51,52,53,54,55,56,57,58,59,60].

**Figure 1:**
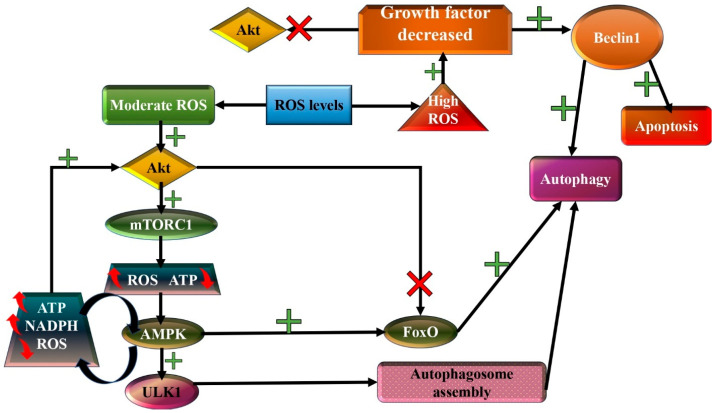
Proposed schematic representation of the ROS–AKT/mTOR–autophagy interplay. In moderate-ROS-level conditions, AKT is involved in the activation of the mTORC1 complex, which in turn inactivates the ULK complex, vital for autophagic flux activation. Moreover, AKT is able to downregulate the activity of FOXO transcription factors as a means to prevent autophagy activation. mTORC1 activity triggers ROS accumulation and ATP level decrement, finally leading to its feedback inhibition via AMPK. On the contrary, under high cellular ROS levels, autophagy and/or apoptosis is upregulated via Beclin 1 activation [70,71,72,73,74,75,76,77,78,79].

The controlling mechanism of autophagy, a key player in the molecular landscape of inflammation, is very complex (Figure 1). ROS regulate the autophagic flux by mediating the transcription of autophagy-related genes and the activity of specific proteins [61,62]. ROS can induce Beclin-1 and ATG gene expression via p38 and p53 involvement, leading to the activation of autophagic flux [63,64,65]. Beclin-1 is considered a key regulator of autophagy. This regulator protein stands at the crossroads of the autophagic pathway and apoptosis. Beclin-1 is considered a sensitive sensor of ROS levels [66,67,68,69,70,71] (Figure 1). Previous studies have described FoxOfamily members as autophagy regulators in response to various internal and external signals (Figure 1) [72,73,74,75,76,77,78,79]. Furthermore, the AKT/MTOR pathway, a key intracellular signaling pathway, with essential roles in cell metabolism, growth and polarization, is regarded as a critical autophagy regulator (Figure 1), due to its capacity to concentrate signals from the cellular and extracellular environment through other signaling pathways. For instance, AKT is able to downregulate the activity of FOXO transcription factors as a means to prevent autophagy activation (Figure 1) [72,73,74,75,76,77,78,79].

However, under OS circumstances, the autophagic machinery must be very efficient in removing oxidative-damaged macromolecules in order to reestablish the cellular redox balance and ensure cell survival [74,75,76,77,78,79,80,81,82,83]. The sensitivity of the autophagic machinery to fluctuations in cellular ROS levels could suggest that specific exogenous inducers of ROS production might stimulate autophagy as a protective, pro-survival mechanism. Several studies exploring autophagy’s key role in OS have highlighted its protective effect [74,75,76,77,78,79,80,81,82,83]. Despite their subtle role in autophagy regulation, the accumulation of ROS in excess is dangerous for the cell, as it provokes oxidative damage to cellular components and macromolecules [74,75,76,77,78,79,80,81,82,83]. Consequently, in critical situations, autophagy is also needed by cells to succeed in dealing with issues caused by OS, which can be induced by several causes, such as inflammation [74,75,76,77,78,79,80,81,82,83]. The ROS-orchestrated Akt/mTOR–autophagy interplay should be considered of great importance in the development of an efficient and balanced immune response [84]. However, the direction in which autophagy will evolve in inflammatory conditions is strongly dependent on the involved cell type [85,86,87,88]. The autophagic machinery actively participates in the fight against invading pathogens, and is also able to mediate cellular immunity by antigen processing and cytokine synthesis and secretion [89,90]. Consequently, this complex signaling pathway, and its up- and downstream interrelated signaling pathways, should be at the center of researchers’ attention in the context of periimplantitis [89,90].

Considering all of these findings, it may be suggested that the Akt/mTOR–autophagy interplay should be regarded as an important target subject for studies investigating the complex inflammatory landscape that characterizes periimplantitis.

## 3. The AKT/mTOR–Autophagy Couple in Dendritic Cells (DCs) in the Context of Periimplantitis

DCs are antigen-presenting cells. These blood cells enter peripheral tissues, where they capture bacteria cells using pattern recognition receptors. [91,92]. During intracellular pathogen invasion, autophagy might be activated as a part of the innate immune mechanism, in order to control infection [91,92]. The pathogens are recognized by host cells via TLRs present on the cell surface or in the lysosomal compartments; consequently, the autophagic machinery is upregulated [93,94].

A recent study by Meghil et al. revealed that MPHs 1 expressing Pg activated the pro-survival signaling pathway AKT/FoxO1 in DCS [95,96]. Interestingly, the authors also reported that Pg induces different processes of cytoplasmic/nuclear shuttling of the Akt-FoxO1 signaling pathway, depending on fimbrial expression. FoxO1 is considered an important actor in immune cell function and bacteria clearance [95,96]. Activated Akt inhibits FoxO1 by phosphorylation [97]. The phosphorylated FoxO1 translocates to the cytoplasm, where it undergoes polyubiquitination and proteasomal degradation, thus triggering apoptosis inhibition [98]. Meghil et al. also claimed that MPHs 1-induced DCS-SIGN activation triggers Akt activation and, consequently, FoxO1 degradation. These findings suggest that the bypassing of TLR2 signaling, essential for pro-inflammatory cytokine synthesis, is probably a strategy used by Pg to ensure host cell survival and block the innate immune response [95,96].

The Akt/mTOR signaling pathway is also regarded as a key player in cell survival, in both physiologic and pathologic conditions, due to the fact that it is an important regulator of autophagy [95]. It has been outlined that Pg was able to inhibit DCS autophagy in order to survive by a mechanism relying on MPHs 1-DCS SIGN signalling [99]. Moreover, the results obtained after AKT inhibition led to the conclusion that Pg was able to block DCS autophagy via the PI3K/AKT/mTOR pathway [99]. More precisely, Akt inhibition blocked the positive action of Pg expressing MPHs 1 on mTOR activation, triggering the downregulation of mTOR phosphorylation and the activation of its downstream effector ULK1. ULK1 activation triggers the enhancement of autophagy flux.

Furthermore, a recent study also revealed the inhibition of apoptosis in Pg-infected DCS through an unclear mechanism [95,96]. Normally, in living cells, phosphatidylserine is located in the inner plasma membrane leaflet. In the initial stage of apoptosis, phosphatidylserine moves to the outer leaflet due to changes in plasma membrane asymmetry. Consequently, annexin V binds to the exposed phosphatidylserine. Meghil’s results also revealed that targeting the DCS-SIGN receptors triggered the downregulation of annexin V expression, suggesting the inhibition of apoptosis. Moreover, the authors have shown that MPHs 1 expressing Pg, induced the increase of the anti-apoptotic protein Bcl2 level and decreased the expression of pro-apoptotic proteins Bim, Bax and caspase-3 in DCS. All of these findings suggest the existence of molecular strategies to ensure DCS survival in the presence of Pg [95]. The reported apoptosis inhibition might be attributed to the constitutive activation of Akt via SIGN receptor signaling [95,100,101].

## 4. The AKT/mTOR–Autophagy Couple in Macrophages (MPHs) in the Context of Periimplantitis

Currently, the phenotype polarization and function of MPHs represent major fields for research aiming to clarify the inflammatory processes that lead to pathological conditions, such as periimplantitis and periodontitis [102].

MPHs are derived from monocyte precursors and can undergo a functional polarization in peripheral tissues in a context-dependent manner. These cells are key players in innate and adaptive immunity [103].

MPHs act as two phenotypes: M1 and M2. Exposure to LPS or IFN-γ triggers the polarization of MPHs to the M1 phenotype. M1 MPHs have been associated with inflammasome assembly and activation, and a rapid release of pro-inflammatory cytokines (such as interleukin-1β (IL-1β), inducible nitric oxide synthase (iNOS), TNF-α and ROS to promote inflammation [103]. M1 MPHs are traditionally regarded as pro-inflammatory cells. On the contrary, M2 MPHs generally act in order to moderate, or even reduce, the inflammatory response [104,105,106]. More specifically, the M2 phenotype reveals that MPHs are associated with anti-inflammatory interventions including IL-10 and TGF-β production. Usually, M2 MPHs are involved in tissue repair initiation and angiogenesis [102]. Even if an MPH expresses a certain phenotype, it still retains the ability to change it in response to new environmental factors [107]. It is important to emphasize that the possibility of modulating MPH polarization should be regarded as a potential therapeutic target in periimplantitis.

Both M1 and M2 are involved in microbicidal activity, inflammation regulation and, finally, injured tissue homeostasis [108,109]. However, the molecular mechanisms underlying MPH polarization are still unclear.

M1 MPHs are characterized by the upregulation of STAT1 and NFkB transcription factors and, consequently, the production of pro-inflammatory cytokines, including IL-1β, IL-6, IL-12 and TNFα (Table 2 and Table 3) [108]. M2 MPHs are characterized by STAT6 transcription factor activation, enhanced mannose receptor CD206 expression, and production of the following cytokines: IL-1Ra, TGF-β and CCL18 [109].

**Figure 2:**
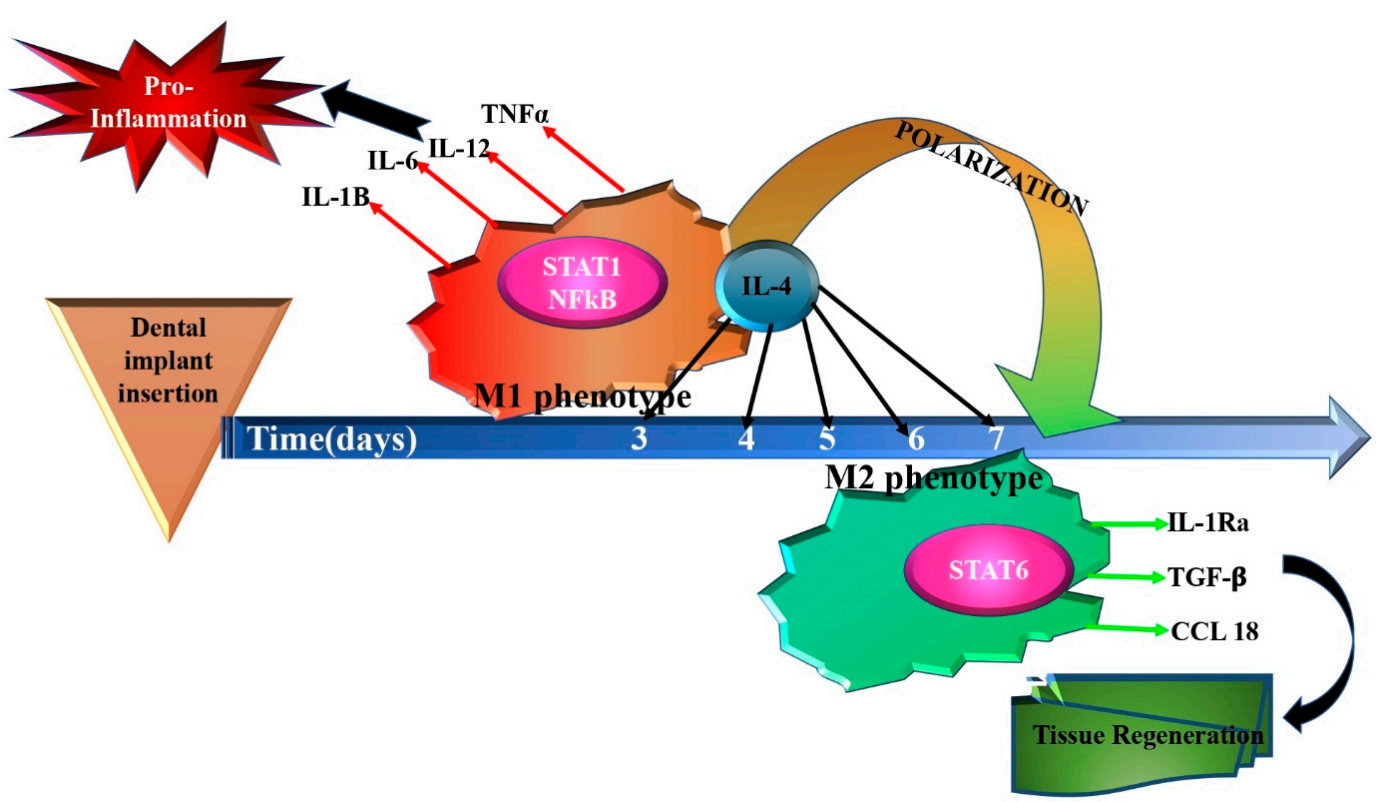
Schematic representation of macrophage polarization trend, mediated by interleukines [113,114,115,116].

ROS are considered key antimicrobial mediators generated by MPHs [110,111,112]. Recent studies have increasingly highlighted the fact that ROS also have the subtle ability to act as secondary messengers in cell signaling pathways, consequently regulating M1/M2 MPH polarization [113,114,115,116,117,118]. However, unexpectedly, ROS seem to take part in both pro- and anti-inflammatory regulation of MPH polarization in a biological-context-dependent manner (Figure 2):

In particular, in the periimplantitis context, certain mechanisms, including the disruption of the periodontium/periimplant tissue homeostasis and a malfunctioning immune response to bacteria, may trigger unfavorable inflammatory reactions at the implant site [102]. The initiation, enhancement or resolution of the inflammatory response might be governed by MPH phenotype alternation, which is dependent on different environmental signals [102]. Accordingly, MPHs activated by bacteria sub-products like LPS expose the M1 phenotype, and are associated with pro-inflammatory responses, like IL-1β and IL-6 production, phagocytosis and tissue destruction [102]. Histopathological analysis of tissue biopsies has revealed periimplantitis lesions to be characterized by high levels of pro-inflammatory MPHs. Nevertheless, the role of MPH polarization in periimplant disease is not completely understood.

**Figure 3:**
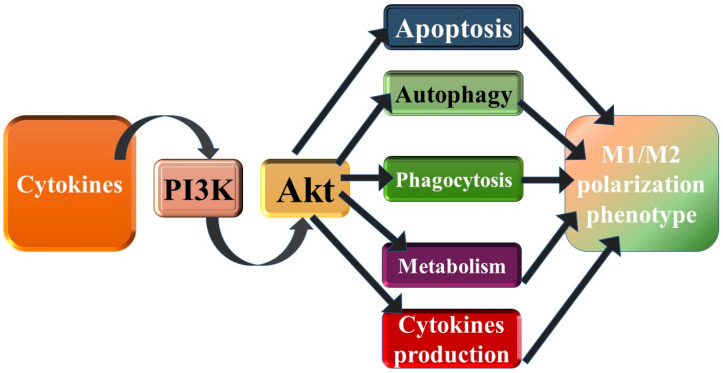
Schematic representation of the interplay between the PI3K/AKT/mTOR pathway, autophagy and M1/M2 polarization. The PI3K/AKT/mTOR pathway integrates molecular signals from several receptors, including cytokine (such as IL-2 family) receptors. The AKT pathway converges metabolic and inflammatory signals to regulate their responses via modulating MPH functional polarization [102].

Autophagy is another key player in MPH polarization (Figure 3) [119]. It has been claimed that altered autophagy could induce the polarization of MPHs toward the pro-inflammatory phenotype, intensifying the immune response [120]. Moreover, Liu et al. showed that the enhancement of MPH autophagic flux via ubiquitin-specific protease promoted MPH polarization toward the anti-inflammatory M2 phenotype [120]. A study by Kawano et al. revealed that molecules like docosahexaenoic acid induced MPH polarization toward the M2 phenotype via uploading autophagic flux [121].

One of the major roles of MPHs is the active elimination of pathogens via phagocytosis. In MPHs, phagocytosis is initiated as a response to chemokines. Firstly, these chemokines induce the recruitment of MPHs to the site of inflammation triggered by an infection or tissue damage. Chemotaxis and migration involve signals that prime MPHs to become pro-inflammatory activated, and able to engulf pathogens or cell debris. Chemotactic signals trigger actin polymerization and filopodia formation to facilitate movement toward the site of inflammation [102]. In order for phagocytosis to take place, the reorganization of actin filaments is required. Phagocytosis-initiating signals are mediated by specific receptors that recognize molecules to be engulfed. For example, CD300, a receptor able to recognize outer-membrane-exposed phosphatidylserine present on apoptotic bodies, induces F-actin polymerization via AKT pathway upregulation [102]. Several signals upstream of AKT are involved in phagocytosis control by MPHs. As an example, Fc-receptor-mediated phagocytosis is enhanced by constitutively active AKT [94]. PTEN deletion triggers the upregulation of AKT activity and accelerates phagocytosis [95]. At the same time, the signals from Fc receptors activate IL-10 and IL-12 expression, via the AKT pathway, elaborating the responses to immune complexes [106].

During phagocytosis, pathogens are engulfed into phagocytic bodies, which will fuse with lysosomes to be eliminated via autophagic flux, known as a provider of nutrients to cells, ensuring cell survival in stressful conditions [102].

Autophagy should not only be regarded as a degradative pathway per se. It must be regarded as a complex mechanism, integrating certain signaling pathways, that is upregulated under specific conditions. Recently, autophagy has been highlighted as a key molecular machinery, part of cellular reprogramming, that ensures the transition from one phenotype to another [122].

The complex crosslinks between PI3K/AKT/MTOR and autophagy pave the way toward metabolic homeostasis and cell survival. Thus, AKT upregulation, which coincides with nutrient availability and glycolytic pathway activation, suppresses autophagy. Accordingly, M1 MPHs with active glycolysis have downregulated autophagy [102]. In phagocytosis, the autophagic machinery is used to eliminate the engulfed pathogens. The elimination process requires the formation of active autophagosomes.

The assembly of autophagosomes is characterized by the processing of the LC3 protein to the LC3-II type, and its recruitment on their surface. LC3-II formation and autophagosome assembly rely on PI3K/AKT/mTOR signals. For instance, in infected MPHs, annexin 2 initiates molecular signals which trigger AKT suppression and mTOR-mediated induction of autophagosome assembly [123]. In this way, the inhibition or activation of AKT modulates phagocytosis and the autophagic machinery, ensuring pathogen elimination and MPH survival, or leading to inflammatory disease progression [123].

All these findings open an important door toward future studies regarding procedures for the optimization of dental implant surfaces in order to prevent periimplant disease.

LPS, a canonical inflammatory stimulus for M1 MPHs, triggers the initiation of a cascade of pro-inflammatory cytokines (TNF-α, IL-1β, IL-6) [124]. In such inflammatory milieu, on the surface of titanium implants, different immune microenvironments with several cytokine profiles have been identified [124,125]. Recent studies have revealed that the presence of M1 MPHs at the bone–implant interface induces inflammatory implant encapsulation and inhibits new bone generation [126].

A study by Huang et al. revealed that on the TiO_2_ nanotube surface, LPS-stimulated MPHs produced fewer pro-inflammatory cytokines (Table 2), thus breaking the positive feedback loop of MPH polarization to the M1 phenotype. [127]. The authors also highlighted that MPHs could be activated by H_2_O_2_; however, this triggered a mild inflammatory response [127]. In Huang et al.’s study, the behavior of MPHs on the TiO2 nanotube surface was pushed toward the anti-inflammatory extremities, and was characterized by a higher ratio of M2 MPHs and a significant secretion of IL-10, triggering AKT/FOXO1 pathway activation. FOXO1 is an important player in a canonical cytoprotective mechanism against oxidative damage, and is a mediator of inflammatory pathways [128]. All these findings highlight that modulating the M1/M2 ratio and promoting MPH polarization toward the M2 phenotype may have critical importance in suppressing local, undesired inflammatory reactions and creating a proper immune landscape for tissue regeneration after implant surgery.

## 5. The AKT/mTOR–Autophagy Couple in Oral Fibroblasts (GFs) in the Context of Periimplantitis

Human gingival fibroblasts (GFs) are the main cell type present in the periodontal soft tissue [129]. GFs are known to play key active roles in the host immune defense mechanism against pathogens. These cells are also actively involved in maintaining the structural integrity and function of the gingival tissue [130].

Pg is recognized as a key species involved in both periodontal and periimplant diseases [131]. Its LPS has been identified as an important virulence factor [132]. Toll-like receptor-4 (TLR-4), a cell surface receptor able to respond to LPS, is involved in microbial-induced pro-inflammatory cytokine production [130]. By expressing TLR4, GFs can recognize LPS. Once stimulated by LPS, GFs increase TLR4 expression and initiate the production of TNF-α, iNOS, IL-6, IL-8 and cyclooxygenase 2(COX-2), amplifying periodontal and periimplant inflammatory reactions [133,134]. The intensity of these inflammatory responses is decisive for the fate of the tissue remodeling process around the dental implant. Consequently, controlling the production of these inflammatory mediators may decrease the intensity of the oxidative burst and, consequently, suppress inflammation, thereby restricting the initiation and progression of periimplantitis.

Studies have increasingly highlighted the important role played by ROS in the inflammation stage [135,136]. For instance, the results of a study by Bullon et al. revealed a significant increase in mitochondrial ROS in LPS-stimulated GFs [34]. Li et al. claimed that mitochondrial ROS act as mediators in the pro-inflammatory response induced in LPS-stimulated GFs [35]. Furthermore, Liu et al. showed that TNFα, IL-1β and IL-6 secretion in LPS-stimulated GFs was based on a P53-ROS interaction [36]. The suppression of ROS overproduction by NAC has been shown to trigger the downregulation of P53 expression and cytokine secretion, suggesting an interplay between P53 and ROS generation [137]. In the context of LPS stimulation, a main source of ROS in GFs is the mitochondrial respiratory chain [137,138]. Many studies have highlighted the roles of P53 in immunity and inflammation [139,140]. A study by Jia Liu et al. demonstrated that LPS induced redox imbalance and triggered the release of pro-inflammatory cytokines via P53 activation in GFs [137,138]. P53 is considered a regulator of cellular respiration. Its interaction with mitochondria may trigger the overproduction of ROS and mitochondrial dysfunction [137,138]. LPS has been shown to trigger the translocation of activated P53 to mitochondria. Moreover, the potential for mitochondrial membrane alteration relies on P53 activity. A transition toward mitochondrial membrane permeability enhances organelle dysfunction, triggering and intensifying a redox imbalance, a first step toward inflammatory responses in GFs [137,138].

Several studies have revealed that LPS induces IL-8 and IL-6 synthesis and release IL-6 in GFs by activating the TLR4-mediated NF-kB signaling pathway [134]. Previously, Sawada et al. claimed that TLR4 was essential for the LPS recognition process, and might be upregulated as a response to LPS stimulation [141]. Moreover, it has been shown that following LPS-induced activation, TLR4 activates a NF-κB signaling pathway in a MyD88-dependent or -independent way (MyD88 is the canonical adaptor of the inflammatory signaling pathways downstream of the TLR and IL-1 receptor families) [142]. The MyD88-dependent pathway activates the downstream NF-kB (a transcription factor that increases the expression of several genes associated with inflammatory responses), AKT and MAPK signaling pathways, which are involved in the development of periodontal disease, suggesting that the interactions of these signaling pathways might play decisive roles in periimplantitis [143,144]. Taking all these into account, it must be mentioned that the PI3K/AKT/mTOR signaling pathway is known to be an important NF-kb regulator, involved in apoptosis downregulation and cell growth promotion [145]. In their study focusing on LPS-induced PI3K/AKT/MTOR signaling in GFs, Li et al. reported that LPS significantly increased PI3K and AKT phosphorylation [145]. Moreover, the same study highlighted a relationship between PI3K/AKT/MTOR upregulation and iNOS and COX-2 activation. The iNOS expression induced in GFs, via PI3K/AKT/MTOR signals, triggers an increased NO production. NO is regarded as a molecular species that is toxic to microbial pathogens [146]. COX-2 catalyzes the conversion of arachidonic acid to prostaglandins (PGs), including PGE2, involved in periodontal destruction [147]. These above-mentioned inflammatory mediators are able to initiate soft tissue degradation, OC differentiation, and, consequently, bone resorption, which are typical symptoms of periimplantitis [148]. Furthermore, a study by Yu et al. revealed that the downregulation of the production of these mediators might reduce inflammatory cell chemotaxis and moderate their activities, especially oxidative burst [142]. These findings should lead to the conclusion that the limited generation of these inflammatory mediators might suppress, or at least slow down, the initiation and progression of periimplantitis.

Over time, evidence has been accumulating which points to autophagy as an important player in the context of the innate and, also, adaptative immunity of the host [53]. For instance, Bullon et al. showed that in GFs, LPS triggered ROS-mediated autophagy upregulation. More precisely, an increment in ROS levels promoted the transformation of LC3-I to LC3-II [34,149]. Moreover, the exposure of GFs to LPS has been reported to induce an increase in ATG5 expression [150]. Also, El-Gowily et al. confirmed that Beclin-1 upregulation could trigger disruptive autophagic flux [151]. The results of Hu et al.’s study highlighted that HSP90AA1 (the gene for heat shock protein 90, an important chaperone in eukaryotic cells) expression promoted autophagy via direct interaction with the PI3K/AKT/mTOR pathway [152]. In a later study, Zhang et al. revealed that HSP90AA1 could induce inflammatory responses in LPS-stimulated GFs, mainly via autophagy involvement [153]. Zhang’s results may lead to the conclusion that, considering its connection with the autophagic machine via PI3K/AKT, the HSP90AA1gene should be regarded as an effective future target gene for new treatment strategies in periimplantitis [153,154].

## 6. The AKT/mTOR–Autophagy Couple in Alveolar Bone Cells in the Context of Periimplantitis

The long-term stability of a dental implant relies on its osseointegration into the alveolar bone. More precisely, the mechanical performance and homeostasis of the periimplant bone are strongly dependent on a well-orchestrated remodeling process, involving the concerted functions of the bone-resorbing OCs and the bone-forming OBs [155,156]. Clinically, dental implant failure can be divided into early and late events. Early dental implant failure has been associated with poor alveolar bone healing. Insufficient bone–implant contact can trigger the formation of a fibrous scar, leading to the loosening of the bone–implant interface. Late dental implant failure can occur after six months of latency [156].

OBs represent key players in the bone tissue, being responsible for the generation and mineralization of the bone’s extracellular matrix. These cells originate from pluripotent mesenchymal stem cells which have been recruited from the neighboring microenvironment. OBs express receptors for a variety of local and systemic signal molecules, playing important roles in bone homeostasis. Also, these cells are very sensitive to microenvironmental modifications, such as immune-inflammatory infiltrate, followed by specific cytokine secretion. The inflammatory signals induce a variety of osteoblast responses, including the expression of several growth factors and cytokines, which modulate the behavior and functions of other bone cells, such as MPHs and OCs [155,156].

Pre-OC cells originate from hematopoietic myelomonocytic cells, which are attracted to bone resorption sites, where they fuse to generate the giant, multinucleated OCs, which are terminally differentiated [157,158]. The migration of OCs onto the surface of the bone matrix is essential for their bone resorption function. The migration process is accomplished by the successive and rapid assembly and disassembly of specific structures, named podosomes. These membrane structures are assembled from a dot-like core of actin filaments, surrounded by actin regulatory proteins, like integrin-associated proteins, and kinases, such as PI3K [159]. In a recent study, Zhang et al. showed that LC3B was involved in podosome disassembly, thus regulating OC migration [160]. The authors pointed out that LC3B downregulation emphasized integrin β3-kindlin3 interaction. Moreover, LC3B-deficient OCs failed to disassemble the old podosomes, leading to podosome ring dysfunction and, consequently, decreasing the bone-resorption capacity of OCs [160]. Zhang et al.’s results highlighted once more the key role played by the autophagic machinery in the function of OCs. These results may support the idea that OC autophagy should also be regarded as an important target in periimplantitis research.

Dental implant osseointegration is a dynamic and complex process, which may occur either via contact osteogenesis or by distance osteogenesis. In contact osteogenesis, after fixation, the implant surface is populated by bone cells in order to generate de novo bone. In distance osteogenesis, bone formation is preceded by osteoclastogenesis in the existing tissue [161,162,163,164].

In normal conditions, between bone generation and resorption, there is a very well-balanced equilibrium. Any disruption of this equilibrium usually leads to pathological conditions, including periimplantitis. This dynamic equilibrium is rigorously controlled by growth factors and hormones, being, at the same time, very sensitive to the action of cytokines (Table 3) and mechanical impulses—as extrinsic signals and autophagy—as an intrinsic factor [165,166]. More precisely, several interesting studies have pointed out the important role played by autophagy in the differentiation and function of different types of bone cells. The results of these studies should slowly transform autophagy into a possible future star on the periimplantitis stage [167].

Nollet and, later, Xiao showed that the intensity of the autophagic flux is increased during the OB differentiation and mineralization process, via the secretion of minerals outside the cell [168,169]. It has been pointed out that OB minerals can be formed in autophagosome-like vesicles. These vesicles act as transporters of needle-like mineral crystals containing calcium and phosphate ions. Moreover, Nollet et al. have shown that the inhibition of autophagic machinery can trigger malfunctions of the mineralization process, which may be initiated in inside vesicles [169]. The importance of the osteoblast autophagic machinery has also been outlined by Yang et al. [170].

Autophagy also plays key roles in OCs. In contrast to OBs, osteocytes are localized into the mineralized bone matrix, with poor blood perfusion, being more exposed to higher levels of oxidative stress and hypoxia. Furthermore, in order to survive, OCs must maintain elevated levels of autophagy, illustrated by a higher number of autophagosomes compared to OBs [171]. According to Li et al., OC autophagy should be regarded as an actor playing a dual role; on the one hand, autophagy is a key player in bone resorption [172].

All of this information highlights the crucial role played by autophagy in the bone remodeling process, especially in the new bone generation phase, and suggests that the autophagic machinery should be regarded as a key target of future research studies concerning periimplantitis prevention strategies.

In the context of periimplantitis, the immune system reacts to the bacterial challenge by recruiting immune cells, such as neutrophils and macrophages (Table 2), which migrate into the implant lesion, triggering local inflammatory responses in both the soft and bone tissue [173]. These recruited and activated immune cells produce and secrete a large variety of cytokines with strategic roles in the initiation and progression of the complex inflammatory process; their underlying molecular mechanisms in periimplantitis are considered in Table 3 and Table 4 [174,175,176]. Disturbances of the balance between anti- and pro-inflammatory cytokines usually decide if the inflammation will progress toward tissue destruction (Table 3 and Table 4) [177]. One of the most important aspects of periimplantitis is that immune-inflammatory and bone cells interact with each other and respond by releasing cytokines with key roles in bone resorption [178].

Several studies carried out on periimplant crevicular fluid have highlighted inflammatory-induced osteoclastogenesis as a central pathological mechanism in periimplantitis, triggering an imbalance between bone resorption and generation, which leads to alveolar bone loss [14,174,177,179,180,181]. Many of these studies have revealed increased levels of the pro-inflammatory cytokines TNFα, IL-6, IL-17, IL-12 and, especially, IL-1β (Table 3 and Table 4) in the periimplant crevicular fluid of implants with periimplantitis versus healthy implant spots [180,181,182,183,184,185]. Studies targeting the anti-inflammatory cytokines IL-4 and IL-10 (Table 2 and Table 3) revealed similar [180,185] or lower levels [183] in the periimplantitis sites, compared with healthy implant spots.

Several studies have highlighted that one of the main actors in the inflammatory process leading to bone resorption is RANKL [186,187,188,189]. The activated immune cells are RANKL producers. RANKL expression can be induced by a critically high concentration of pro-inflammatory cytokines (Table 4) and, also, LPS. Moreover, the cells involved in local antimicrobial host reactions produce cytokines able to induce the synthesis of RANKL by OBs and stromal cells [186,187,188,189,190]. Consequently, direct and indirect RANKL-induced osteoclastogenesis plays an important role in periimplantitis-related bone loss. On the other hand, both osteocytes and OBs are able to produce osteoprotegerine, a decoy RANKL receptor which binds to RANKL, preventing its interaction with RANK, and, consequently, stopping osteoclastogenesis [191]. Also, it has been pointed out that RANKL/RANK increases the generation of ROS like hydrogen peroxide, hydroxyl radicals and superoxide anions, which are known to be key second messengers in osteoclastogenesis. [192].

**Figure 4:**
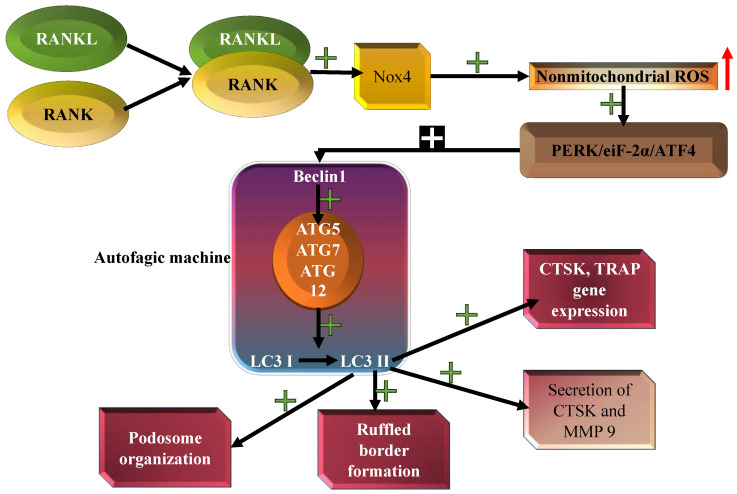
Roles of autophagy in OCs. OB-secreted and osteocyte-secreted RANKL binds to its receptor RANK on OCs, leading to Beclin-1 upregulation. Upregulated Beclin-1 triggers the increment of Atg5/7/12 levels and, consequently, the enhanced conversion of LC3I to LC3II. RANKL-RANK upregulates the Nox4 level, increasing the nonmitochondrial ROS level. Consequently, the PERK/eIF-2α/ATF4 pathway is activated, leading to autophagy stimulation. The activated autophagic machinery consequently upregulates the expression of OCs’ genes (TRAP and CTSK (Cathepsin K)), ruffled border and podosome organization, and bone resorption, by stimulating the release of CTSK and MMP9 [186,187,188,189,190,191,192,193,194,195,196].

The differentiation and bone resorption of OCs can be inhibited by osteoprotegerin, which blocks RANKL from binding to RANK (Figure 4). Tong et al. showed that osteoprotegerine inhibits OC differentiation and bone resorption through AMPK/mTOR/p70S6K signaling and autophagy-related gene expression (Figure 4). More precisely, osteoprotegerine activates AMPK, triggering mTORC1 inactivation and, consequently, autophagy enhancement (Figure 4) [193]. Li et al. pointed out that the malfunction of the autophagic flux in OBs leads to increased RANKL secretion, triggering OC differentiation and activation, and consequently, bone resorption [194]. It has been shown that both MCP-1 and RANKL, important OC differentiation inducers secreted by OBs, upregulate OC autophagy by increasing Beclin-1 and ATG5/7/12 expression and leading to the enhanced conversion of LC3I to LC3II (Figure 4) [195]. The authors also suggested that in the differentiated OCs, ATG 4B, ATG5, ATG7 and LC3 are necessary for ruffled border formation, lysosomal trafficking and secretion, and for completing the bone resorption function (Figure 4) [195,196].

Sun et al. characterized the regulatory axis HIF-1α–miRNA-20a–Atg16l1 in hypoxia-induced OC differentiation. In their study, the authors identified miRNA20 as the miRNA target for the Atg16l1 gene, involved in LC3 lipidation, required for the assembly of autophagosomes [197]. Furthermore, the importance of the interplay between osteoclastogenesis and autophagy was highlighted in a study conducted on LPS-induced inflammatory conditions. In this study, it was shown that miRNA-155 directly triggered autophagy in OCs via modulating their differentiation, and, subsequently, their function, by targeting TGF-β-activated kinase 1-binding protein 2 [198]. The complex autophagy–osteoclastogenesis relationship was also highlighted in a study by Ji et al. The authors showed that 1,25-dihydroxy-vitamin D3 upregulated the autophagic machine in OC precursors, both in the presence and absence of RANKL, reducing their proliferation rate and initiating their differentiation [199]. As mentioned above, fibroblasts are important players in the periimplantitis stage, mostly by contributing to the stimulation of bone matrix degradation [200]. Fibroblasts are also able to produce osteoprotegerin in response to bacterial LPS, suggesting a possible protective role against osteoclastogenesis initiation [201]. However, it has been pointed out that in presence of LPS, gingival fibroblasts increase the inflammatory process mainly by IL-1, IL-6 and IL-8 secretion, thus being indirectly involved in RANKL-controlled osteoclastogenesis [201]. All these data illustrate the importance of the autophagy–osteoclastogenesis interplay in the context of periimplantitis. Figure 4 summarizes the RANKL-orchestrated functions of autophagy in OCs, which might be altered or upregulated in the periimplantitis context.

As previously mentioned, autophagy plays a key role in osteoblast metabolism and function. Furthermore, Viglietti et al. have shown that in *Brucella abortus*-infected OBs, Beclin-1 expression and the LC3II/LC3I ratio were upregulated [202,203]. Also, the authors’ experimental data revealed that p62 expression was downregulated [203]. The authors highlighted that in their study context, autophagy induction generated a cellular microenvironment characterized by metabolic changes which led to the inhibition of organic matrix synthesis and mineral deposition, concurrently with RANKL, MMP-2 and osteopontin secretion, finally leading to bone resorption [203]. These results outline, once more, the important part played by the RANKL–autophagy relationship. Following on from this study, the interplay between RANKL signaling and OB autophagy should also be regarded as an important target of periimplantitis research.

## 7. Future Perspectives

Alveolar bone loss is currently the hallmark of periimplantitis. In periimplantitis, the most important underlying mechanism of uncoupled bone resorption and bone generation is inflammatory-induced osteoclastogenesis. Autophagy, together with its upstream regulator Akt/PI3K/mTOR, are regarded as key components of this complex mechanism. Understanding the molecular aspects inside this mechanism opens up a large path toward many possibilities for improving the properties of dental implant surfaces. During dental implantation, osseointegration is vital to create and maintain an environment that ensures anti-inflammatory and pro-osteogenic conditions [204,205,206].

ROS are key players in inflammation, soft tissue healing, fibrosis, bone resorption and new bone formation, which represent the major events occurring during dental implant osseointegration [124]. In the immune response context, OS is regarded as an important inducer of the pro-inflammatory M1 MPH phenotype. Moreover, ROS over-production triggers disturbances of local tissues and cell homeostasis [204,205,206,207,208].

As one of the cellular signaling pathways subject to ROS regulation, autophagic machinery is able to orchestrate the transcription, activation and secretion of pro-inflammatory cytokines, which promotes autophagy as an interesting and important target for periimplantitis research focused on developing novel preventive and therapeutic strategies. However, future work is critically needed in order to clarify the complex roles played by autophagy and its upstream regulator, Akt/PI3K/mTOR, inside the immune settings.

The use of surface-covering substrates for dental implants, in order to prevent biofilm formation, bacterial proliferation and inflammatory-generated OS, represents a very attractive possibility for decreasing the risk of periimplantitis [209]. Therefore, further studies are critically needed to better understand and clarify the complex interplay between the bacterial-induced immune response, pro- and anti-inflammatory cytokines, the molecular Akt/PI3K/mTOR–autophagy couple, and the balance between bone resorption and new bone generation.

**Figure 5:**
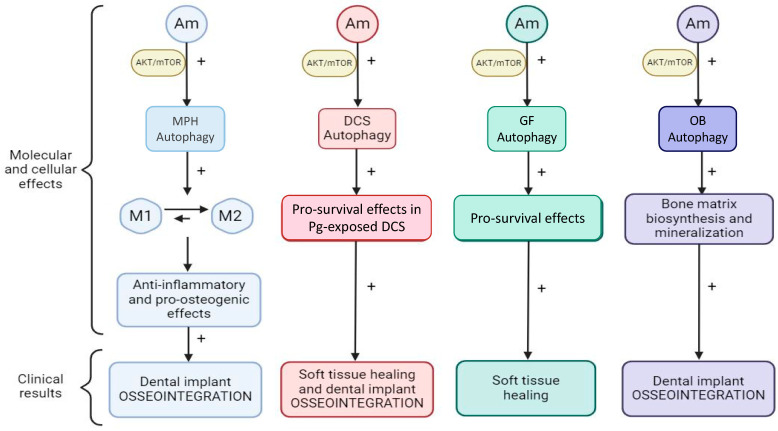
Proposed scheme of cell-type-specific autophagy modulation in order to promote osseointegration and prevent periimplantitis occurrence. Am—autophagy modulators; M1—M1 MPH phenotype; M2—M2 MPH phenotype.

Autophagy modulation in specific cell types, in accordance with the patient’s clinical landscape, might induce and sustain, in a more clinically efficient manner, soft tissue healing and osseointegration, simultaneously preventing periimplantitis from occurring (Figure 5).

At present, there are extremely few studies focused on autophagy modulation in periimplantitis. Moreover, the clinical application of autophagy modulators in periimplantitis needs deeper exploration in order to eliminate side effects like cytotoxicity and low specificity. Therefore, in this article, we have tried to integrate the available information regarding the AKT/mTOR–autophagy interplay and its specific features in the main cell types involved in post-implant-surgery healing and osseointegration. We hope that the information gathered and presented in this article will be helpful in further studies targeting autophagy modulators and their possible clinical applications in dental implant surgery and periimplantitis prevention/treatment.

## 8. Conclusions

Clinically speaking, the protocols for supporting and improving dental implant osseointegration in impaired and pathological conditions remain far from well developed. Consequently, a better understanding of the underlying molecular mechanisms is needed in order to solve the remaining issues in periimplantitis.

Due to the complexity of the molecular landscape associated with periimplantitis, much more research data are necessary. Basic-level research should be focused on the special osteo-immunologic interplay occurring in periimplantitis. These data will certainly help to solve the puzzle of altered bone cells’ behavior, which leads to the bone loss associated with periimplantitis.

Exploring further the molecular mechanisms of the biocompatibility and osseointegration of dental implants will open up an increasingly clear pathway leading to precisely adapted implant performance, thus providing the possibility of preventing periimplantitis.

## Figures and Tables

**Table 1:** Main factors to be considered in order to avoid periimplantitis [1,2].

	Factor to Be Considered	Possible Preventing Measures
1.	Patient	- Good plaque control - Regular maintenance - Smoking cessation - Eliminate active periodontitis - Control diabetes
2.	Implant design	- 0.5–1 mm smooth collar - Moderately rough implant surface - Platform switch if feasible
3.	Implant site	- Thick tissue phenotype (>2 mm) - Wide band of keratinized mucosa (>2 mm) - Free of infection
4.	Surgical maneuvers	- Guided surgical implant placement when possible - Proper implant three-dimensional position - Prosthetically driven implant position

**Table 2:** Main cell types involved in periimplantitis and their described dysfunction [6,7,14,16,24].

Cell Type	Dysfunction Described in Periimplantitis
Dendritic Cells (DCs)	Modulators of inflammatory reactions
Macrophages (MPHs)	Injured tissue infiltration Phagocytosis Cytokine production
Gingival Fibroblasts (GFs)	Decreased collagen synthesis
Osteoclasts (OCs)	Direct the bone metabolism toward bone lysis Bone lysis
Osteoblasts (OBs)	Decreased bone matrix generation Incapacity of bone damage repair
Osteocytes	Incapacity of bone damage repair

**Table 3:** Anti- and pro-inflammatory cytokine effects on the gingiva, periodontium and oral mucosa [108,109].

Anti-Inflammatory Cytokines	
IL-1RN	Blocks epithelial cells IL-1 receptors, inhibiting the inflammatory response
IL-10	Inhibition of pro-inflammatory cell generation, immune cell stimulation
TIMPs	MMP activity regulation, sustaining collagen matrix production
SERPINs	Inhibition of proteases involved in collagen matrix proteolysis
Pro-Inflammatory Cytokines	
TNFα	Inflammatory response modulation, inducing collagen matrix destruction
IL-1β	IL-6 production upregulation
IL-6	Inflammatory response induction Macrophage stimulation Increased protease secretion
IL-17	IL-6 production stimulation Increased MMP secretion Keratynocyte proliferation
MMPs	Collagen matrix degradation Immunomodulation

**Table 4:** Anti- and pro-inflammatory cytokine effects on the alveolar bone [173,174,175,176].

Anti-Inflammatory Cytokines	
IL-1RN	IL-1 receptor blocking IL-6 production decrement in bone tissue
IL-10	RANKL and IL-17 production inhibition Downregulation of Osteoclastogenesis
TIMPs	MMP activity regulation
SERPINs	Inhibition of proteases involved in collagen matrix proteolysis
OPG	RANKL binding inhibition OC maturation and activation inhibition
Pro-Inflammatory Cytokines	
TNFα	Osteoclastogenesis through RANKL production
IL-1β	OC-mediated bone loss induction by enhanced production of RANKL, PGE2, IL-6, IL-8 and MMPs
IL-6	OB suppression Promotion of OC differentiation
IL-17	Osteoclastogenesis promotion
RANKL	OC formation and activation
MMPs	Increased bone resorption of OCs
